# Simple Peptides Stabilize Mighty Membrane Proteins for Study

**DOI:** 10.1371/journal.pbio.0030259

**Published:** 2005-06-21

Cell membranes are largely made of proteins, and membrane proteins account for about a third of all genes. Despite their importance, they are devilishly hard to isolate and stabilize, and therefore are hard to study. The problem lies in their structure: membrane proteins have at least one hydrophobic domain, composed of a stretch of water-repelling amino acids, which holds the protein snugly in the lipid membrane. Purifying such a protein in an aqueous medium makes the hydrophobic parts aggregate, destroying the protein's delicate three-dimensional structure and often disrupting its function. The alternative is to extract the protein with a detergent, a two-headed “Janus” molecule with both hydrophobic and hydrophilic ends. The protein remains surrounded by the hydrophobic ends, while water clusters at the hydrophilic ends, easing the protein out of the membrane and into solution, where it can be studied.

To date, though, relatively few complex membrane proteins have been successfully purified with available detergents. In this issue, Shuguang Zhang and colleagues show that a simple amino acid–based detergent can successfully stabilize the dauntingly large protein complex photosystem I (PS-I), an integral part of the photosynthetic machinery.

The molecule they made, abbreviated A_6_K, links six units of the hydrophobic amino acid alanine to one of the hydrophilic amino acid lysine. The authors used it to stabilize PS-I and then attached the detergent–protein complex to a glass slide, allowed it to dry, and examined the stability of PS-I by testing its fluorescence. Intact PS-I emits red light with a characteristic peak wavelength; as it degrades, this peak subsides and is replaced by another, bluer peak. Even the two best standard detergents did poorly at maintaining the red peak. In contrast, the spectrum after A_6_K extraction was almost a perfect match for the normal one, indicating the complex was largely intact after drying. Furthermore, the complex appeared to remain stable for up to three weeks on the glass slide.

The potential applications of this work are severalfold. PS-I itself remains to be fully characterized, and this stabilization technique offers new means to explore its properties. In addition, an isolated and stabilized form of PS-I may hold some promise as an alternative energy source, since it generates an electric current in sunlight. Perhaps most importantly, the full potential of such simple amino acid–based detergents has only begun to be explored. It is likely that either this one, or others like it, can be used to isolate and stabilize hundreds of other membrane proteins, allowing them to be studied in detail for the first time. [Fig pbio-0030259-g001]

**Figure pbio-0030259-g001:**
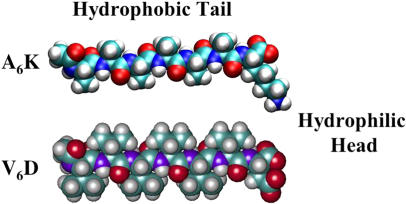
The designed short peptide (protein fragment) detergents look like matches and behave like lipids or oil molecules that repel water at one end but attract water at the other end

